# *catena*-Poly[[silver(I)-μ-1,2-bis­(4,4-dimethyl-4,5-dihydro-1,3-oxazol-2-yl)ethane-κ^2^ *N*:*N*′] perchlorate hemihydrate]

**DOI:** 10.1107/S1600536812016406

**Published:** 2012-04-21

**Authors:** Chun-Wei Yeh, Fu-Chang Huang, Shui-Chuan Lin, Ay Jong, Maw-Cherng Suen

**Affiliations:** a Department of Chemistry, Chung-Yuan Christian University, Jhongli 32023, Taiwan; b Department of Civil and Environmental Engineering, Nanya Institute of Technology, Jhongli 32091, Taiwan; c Department of Chemical and Material Engineering, Nanya Institute of Technology, Jhongli 32091, Taiwan; d Department of Polymer Materials, Vanung University, Jhongli 32061, Taiwan; e Department of Materials and Fibers, Nanya Institute of Technology, Jhongli 32091, Taiwan

## Abstract

In the title coordination polymer, {[Ag(C_12_H_20_N_2_O_2_)]ClO_4_·0.5H_2_O}_*n*_, the Ag^I^ cation is coordinated by two N atoms from two 1,2-bis­(4,4-dimethyl-4,5-dihydro-1,3-oxazol-2-yl)ethane (*L*) ligands in a nearly linear geometry [N—Ag—N = 171.07 (8)°]. The *L* ligand bridges adjacent Ag^+^ cations, forming a polymeric chain running along the *c* axis. The lattice water mol­ecule is situated on a twofold rotation axis, and links to the perchlorate anion *via* an O—H⋯O hydrogen bond. The long Ag⋯O separation of 3.200 (4) Å indicates a weak inter­action between the perchlorate anion and the Ag^I^ cation. Weak C—H⋯O hydrogen bonding occurs between the chain and the lattice water mol­ecule and between the chain and perchlorate anions. Both five-membered rings of the *L* ligand display envelope conformations; in one five-membered ring, the flap C atom is disordered on opposite sides of the ring with occupancies of 0.65 and 0.35.

## Related literature

For background to coordination polymers with organic ligands, see: Kitagawa *et al.* (2004 ▶); Chiang *et al.* (2008 ▶); Yeh *et al.* (2008 ▶, 2009 ▶); Hsu *et al.* (2009 ▶). For related structures, see: Wang *et al.* (2008 ▶, 2011 ▶); Suen *et al.* (2011 ▶).
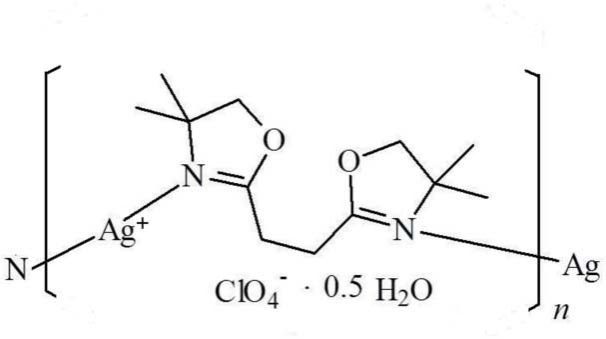


## Experimental

### 

#### Crystal data

[Ag(C_12_H_20_N_2_O_2_)]ClO_4_·0.5H_2_O
*M* *_r_* = 881.26
Monoclinic, 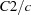

*a* = 25.3322 (19) Å
*b* = 11.2100 (9) Å
*c* = 12.3721 (9) Å
β = 97.917 (1)°
*V* = 3479.9 (5) Å^3^
*Z* = 4
Mo *K*α radiation
μ = 1.34 mm^−1^
*T* = 297 K
0.5 × 0.4 × 0.4 mm

#### Data collection

Bruker APEXII CCD diffractometer
Absorption correction: multi-scan (*SADABS*; Bruker, 2000 ▶) *T*_min_ = 0.753, *T*_max_ = 1.000
9583 measured reflections
3424 independent reflections
2914 reflections with *I* > 2σ(*I*)
*R*_int_ = 0.019

#### Refinement

*R*[*F* ^2^ > 2σ(*F* ^2^)] = 0.029
*wR*(*F* ^2^) = 0.090
*S* = 1.23
3424 reflections
217 parameters
H atoms treated by a mixture of independent and constrained refinement
Δρ_max_ = 0.49 e Å^−3^
Δρ_min_ = −0.41 e Å^−3^

### 

Data collection: *APEX2* (Bruker, 2010 ▶); cell refinement: *SAINT* (Bruker, 2010 ▶); data reduction: *SAINT*; program(s) used to solve structure: *SHELXS97* (Sheldrick, 2008 ▶); program(s) used to refine structure: *SHELXL97* (Sheldrick, 2008 ▶); molecular graphics: *DIAMOND* (Brandenburg, 2010 ▶); software used to prepare material for publication: *SHELXL97*.

## Supplementary Material

Crystal structure: contains datablock(s) I, global. DOI: 10.1107/S1600536812016406/xu5506sup1.cif

Structure factors: contains datablock(s) I. DOI: 10.1107/S1600536812016406/xu5506Isup2.hkl

Additional supplementary materials:  crystallographic information; 3D view; checkCIF report

## Figures and Tables

**Table 1:** Hydrogen-bond geometry (Å, °)

*D*—H⋯*A*	*D*—H	H⋯*A*	*D*⋯*A*	*D*—H⋯*A*
O7—H7*C*⋯O3	0.87 (5)	2.06 (5)	2.926 (4)	175 (4)
C4—H4*C*⋯O7^i^	0.97	2.47	3.379 (4)	156
C5—H5*B*⋯O5^ii^	0.97	2.39	3.289 (5)	153

Symmetry codes: (i) 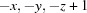; (ii) 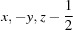.

**Fig. 1.**
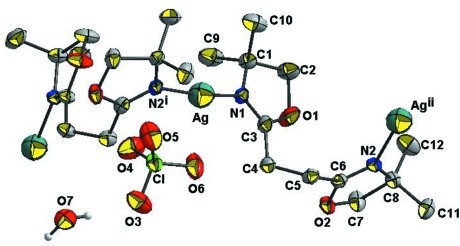
A portion of the one-dimensional chain. Ellipsoids are drawn at 30% probability level, and all the H atoms of L are omitted for clairty. Symmetry codes: (i) x,-y,z + 1/2; (ii) x,-y,z - 1/2.

**Fig. 2.**
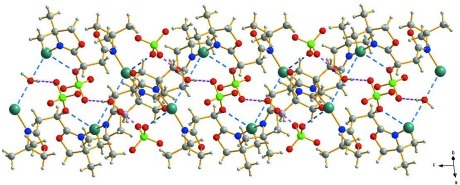
The packing diagram shows the Ag···O interactions and O—H···O hydrogen bonds among the one-dimensional chains.

## supplementary crystallographic information

### Comment

The synthesis of metal coordination polymers has been a subject of intense research due to their interesting structural chemistry and potential applications in gas storage, separation, catalysis, magnetism, luminescence, and drug delivery (Kitagawa *et al.*, 2004). Roles of anion, solvent and ligand comformations in self-assembly of coordination complexes containing polydentate nitrogen ligands are very intersting (Chiang *et al.*, 2008; Yeh *et al.*, 2008; Hsu *et al.*, 2009; Yeh *et al.*, 2009). The d^10^ metal complexes containing 1,4-bis(4,5-dihydro-2-oxazolyl)benzene ligands (*L'*) have been reported, which show various two-dimensional networks (Wang *et al.*, 2008, Wang *et al.*, 2011 and Suen *et al.*, 2011). The Ag^+^ cations are coordinated with two N atoms from two 1,2-bis(4,4-dimethyl-4,5-dihydrooxazol-2-yl)ethane (*L*) ligands (Fig. 1). The Ag···Ag distance separated by the bridging *L* ligands is 6.685 (1) Å, while the bridging *L* ligand adopts *gauche* conformation with C3—C4—C5—C6 trosion angle 67.43 (35) °. The one-dimensional polymeric chains are interlinking through Ag···O interactions [3.045 (1) and 3.199 (4) Å] and O—H···O hydrogen bonds between the Ag^+^ cations, latice water molecules and ClO_4_^-^ anions in the crystal structure (Fig. 2, Tab.1). The C2 atom of the the dihydrooxazol-2-yl group is disordered over two sites with occupancy factors of 0.65 and 0.35.

### Experimental

An aqueous solution (5.0 ml) of AgClO_4_ (1.0 mmol) was layered carefully over a methanolic solution (5.0 ml) of 1,2-bis(4,4-dimethyl-4,5-dihydrooxazol-2-yl)ethane (1.0 mmol) in a tube. Colourless crystals were obtained after several weeks. These were washed with methanol and collected in 68.2% yield.

### Refinement

H atom of the water molecule, H7C, was located in the difference electron density map and refined isotropically, while the other H atoms were constrained to ideal geometries, with C—H = 0.96 (methyl) or 0.97 (methylene) Å and *U*_iso_(H) = 1.2*U*_eq_(C). The C2 atom of the the dihydrooxazol-2-yl ring is disordered over two sites with occupancy factors of 0.65 and 0.35.

### Crystal data

**Table:**

[Ag(C_12_H_20_N_2_O_2_)]ClO_4_·0.5H_2_O	*F*(000) = 1784
*M**_r_* = 881.26	*D*_x_ = 1.682 Mg m^−^^3^
Monoclinic, *C*2/*c*	Mo *K*α radiation, λ = 0.71073 Å
Hall symbol: -C 2yc	Cell parameters from 3424 reflections
*a* = 25.3322 (19) Å	θ = 1.6–26.0°
*b* = 11.2100 (9) Å	µ = 1.34 mm^−^^1^
*c* = 12.3721 (9) Å	*T* = 297 K
β = 97.917 (1)°	Block, colourless
*V* = 3479.9 (5) Å^3^	0.5 × 0.4 × 0.4 mm
*Z* = 4	

### Data collection

**Table:**

Bruker APEXII CCD diffractometer	3424 independent reflections
Radiation source: fine-focus sealed tube	2914 reflections with *I* > 2σ(*I*)
Graphite monochromator	*R*_int_ = 0.019
φ and ω scans	θ_max_ = 26.0°, θ_min_ = 1.6°
Absorption correction: multi-scan (*SADABS*; Bruker, 2000)	*h* = −31→29
*T*_min_ = 0.753, *T*_max_ = 1.000	*k* = −8→13
9583 measured reflections	*l* = −15→13

### Refinement

**Table:**

Refinement on *F*^2^	Primary atom site location: structure-invariant direct methods
Least-squares matrix: full	Secondary atom site location: difference Fourier map
*R*[*F*^2^ > 2σ(*F*^2^)] = 0.029	Hydrogen site location: inferred from neighbouring sites
*wR*(*F*^2^) = 0.090	H atoms treated by a mixture of independent and constrained refinement
*S* = 1.23	*w* = 1/[σ^2^(*F*_o_^2^) + (0.050*P*)^2^] where *P* = (*F*_o_^2^ + 2*F*_c_^2^)/3
3424 reflections	(Δ/σ)_max_ = 0.001
217 parameters	Δρ_max_ = 0.49 e Å^−^^3^
0 restraints	Δρ_min_ = −0.41 e Å^−^^3^

### Special details

**Table:**

Geometry. All e.s.d.'s (except the e.s.d. in the dihedral angle between two l.s. planes) are estimated using the full covariance matrix. The cell e.s.d.'s are taken into account individually in the estimation of e.s.d.'s in distances, angles and torsion angles; correlations between e.s.d.'s in cell parameters are only used when they are defined by crystal symmetry. An approximate (isotropic) treatment of cell e.s.d.'s is used for estimating e.s.d.'s involving l.s. planes.
Refinement. Refinement of *F*^2^ against ALL reflections. The weighted *R*-factor *wR* and goodness of fit *S* are based on *F*^2^, conventional *R*-factors *R* are based on *F*, with *F* set to zero for negative *F*^2^. The threshold expression of *F*^2^ > σ(*F*^2^) is used only for calculating *R*-factors(gt) *etc*. and is not relevant to the choice of reflections for refinement. *R*-factors based on *F*^2^ are statistically about twice as large as those based on *F*, and *R*- factors based on ALL data will be even larger.

### Fractional atomic coordinates and isotropic or equivalent isotropic displacement parameters (Å^2^)

**Table:**

	*x*	*y*	*z*	*U*_iso_*/*U*_eq_	Occ. (<1)
Ag	0.113477 (9)	−0.11300 (2)	0.375689 (18)	0.05407 (11)	
Cl	0.10569 (3)	0.21893 (7)	0.56478 (6)	0.0582 (2)	
O1	0.15659 (9)	0.1990 (2)	0.2086 (2)	0.0816 (8)	
O2	0.05890 (9)	0.35885 (18)	0.09898 (16)	0.0569 (5)	
O3	0.05412 (12)	0.2480 (4)	0.5918 (3)	0.1148 (11)	
O4	0.14340 (13)	0.2630 (3)	0.6480 (3)	0.1168 (11)	
O5	0.11311 (18)	0.0949 (3)	0.5528 (3)	0.1163 (13)	
O6	0.11177 (17)	0.2774 (3)	0.4667 (2)	0.1184 (12)	
O7	0.0000	0.1184 (3)	0.7500	0.0834 (11)	
N1	0.14851 (8)	0.0336 (2)	0.30542 (17)	0.0438 (5)	
N2	0.08939 (8)	0.26102 (19)	−0.03635 (17)	0.0431 (5)	
C1	0.20723 (11)	0.0540 (3)	0.3142 (2)	0.0542 (7)	
C2	0.2107 (4)	0.1392 (8)	0.2200 (10)	0.075 (3)	0.65
H2B	0.2392	0.1965	0.2375	0.089*	0.65
H2C	0.2158	0.0966	0.1540	0.089*	0.65
C2'	0.2083 (9)	0.1825 (15)	0.2631 (19)	0.094 (7)	0.35
H2'A	0.2168	0.2422	0.3195	0.113*	0.35
H2'B	0.2343	0.1870	0.2126	0.113*	0.35
C3	0.12504 (12)	0.1175 (2)	0.2487 (2)	0.0503 (7)	
C4	0.06708 (11)	0.1381 (3)	0.2236 (2)	0.0531 (7)	
H4B	0.0582	0.2111	0.2591	0.064*	
H4C	0.0485	0.0731	0.2537	0.064*	
C5	0.04721 (11)	0.1478 (3)	0.1008 (2)	0.0522 (7)	
H5B	0.0586	0.0777	0.0644	0.063*	
H5C	0.0086	0.1487	0.0901	0.063*	
C6	0.06680 (10)	0.2559 (2)	0.0495 (2)	0.0445 (6)	
C7	0.08686 (15)	0.4491 (3)	0.0448 (2)	0.0649 (8)	
H7A	0.1194	0.4730	0.0903	0.078*	
H7B	0.0646	0.5190	0.0282	0.078*	
C8	0.09930 (11)	0.3892 (2)	−0.0602 (2)	0.0469 (6)	
C9	0.22808 (15)	0.0913 (4)	0.4296 (3)	0.0954 (14)	
H9A	0.2305	0.0226	0.4764	0.143*	
H9B	0.2042	0.1485	0.4544	0.143*	
H9C	0.2627	0.1264	0.4313	0.143*	
C10	0.23499 (15)	−0.0574 (4)	0.2842 (4)	0.0904 (12)	
H10A	0.2245	−0.1234	0.3259	0.136*	
H10B	0.2729	−0.0466	0.2998	0.136*	
H10C	0.2254	−0.0734	0.2078	0.136*	
C11	0.06137 (15)	0.4279 (3)	−0.1592 (3)	0.0712 (9)	
H11A	0.0701	0.3873	−0.2227	0.107*	
H11B	0.0644	0.5125	−0.1691	0.107*	
H11C	0.0255	0.4086	−0.1487	0.107*	
C12	0.15668 (16)	0.4067 (4)	−0.0786 (4)	0.0841 (12)	
H12A	0.1800	0.3805	−0.0153	0.126*	
H12B	0.1629	0.4896	−0.0914	0.126*	
H12C	0.1635	0.3610	−0.1409	0.126*	
H7C	0.0141 (19)	0.158 (4)	0.701 (4)	0.110 (16)*	

### Atomic displacement parameters (Å^2^)

**Table:**

	*U*^11^	*U*^22^	*U*^33^	*U*^12^	*U*^13^	*U*^23^
Ag	0.05656 (17)	0.04575 (16)	0.06076 (17)	−0.00549 (9)	0.01119 (11)	0.01769 (9)
Cl	0.0756 (5)	0.0481 (4)	0.0535 (4)	−0.0027 (3)	0.0185 (3)	−0.0029 (3)
O1	0.0547 (13)	0.0750 (16)	0.1129 (19)	−0.0111 (11)	0.0034 (12)	0.0533 (15)
O2	0.0834 (14)	0.0417 (11)	0.0493 (11)	0.0071 (10)	0.0223 (10)	−0.0064 (9)
O3	0.082 (2)	0.141 (3)	0.129 (2)	−0.0065 (18)	0.0404 (18)	−0.020 (2)
O4	0.104 (2)	0.107 (3)	0.127 (2)	0.0042 (18)	−0.0311 (19)	−0.028 (2)
O5	0.199 (4)	0.0521 (16)	0.111 (2)	−0.0091 (18)	0.066 (3)	−0.0039 (15)
O6	0.202 (4)	0.083 (2)	0.0810 (17)	0.033 (2)	0.059 (2)	0.0225 (16)
O7	0.095 (3)	0.059 (2)	0.097 (3)	0.000	0.017 (2)	0.000
N1	0.0465 (11)	0.0394 (12)	0.0454 (11)	−0.0039 (9)	0.0063 (9)	0.0062 (10)
N2	0.0463 (12)	0.0379 (11)	0.0450 (11)	0.0029 (9)	0.0057 (9)	−0.0084 (9)
C1	0.0448 (15)	0.0487 (16)	0.0687 (18)	−0.0047 (13)	0.0063 (13)	0.0079 (14)
C2	0.044 (3)	0.069 (6)	0.111 (7)	−0.010 (4)	0.014 (4)	0.024 (4)
C2'	0.071 (9)	0.056 (10)	0.15 (2)	−0.015 (8)	−0.017 (12)	0.047 (10)
C3	0.0490 (15)	0.0481 (17)	0.0546 (16)	−0.0082 (12)	0.0095 (12)	0.0119 (13)
C4	0.0485 (15)	0.0534 (17)	0.0589 (17)	0.0008 (13)	0.0123 (12)	0.0124 (14)
C5	0.0514 (15)	0.0446 (15)	0.0602 (17)	0.0014 (12)	0.0066 (12)	0.0046 (13)
C6	0.0457 (14)	0.0398 (14)	0.0465 (14)	0.0072 (11)	0.0009 (11)	−0.0027 (12)
C7	0.102 (3)	0.0403 (16)	0.0546 (17)	−0.0028 (16)	0.0193 (16)	−0.0061 (14)
C8	0.0525 (15)	0.0429 (15)	0.0454 (14)	−0.0048 (11)	0.0074 (12)	−0.0066 (11)
C9	0.058 (2)	0.124 (4)	0.098 (3)	0.002 (2)	−0.0095 (19)	−0.042 (3)
C10	0.062 (2)	0.099 (3)	0.112 (3)	0.006 (2)	0.017 (2)	−0.029 (3)
C11	0.096 (3)	0.058 (2)	0.0557 (18)	0.0048 (19)	−0.0021 (17)	0.0067 (16)
C12	0.066 (2)	0.084 (3)	0.105 (3)	−0.0240 (19)	0.023 (2)	−0.016 (2)

### Geometric parameters (Å, º)

**Table:**

Ag—N1	2.111 (2)	C3—C4	1.476 (4)
Ag—N2^i^	2.120 (2)	C4—C5	1.537 (4)
Cl—O4	1.394 (3)	C4—H4B	0.9700
Cl—O6	1.406 (3)	C4—H4C	0.9700
Cl—O5	1.413 (3)	C5—C6	1.484 (4)
Cl—O3	1.430 (3)	C5—H5B	0.9700
O1—C3	1.352 (3)	C5—H5C	0.9700
O1—C2'	1.40 (2)	C7—C8	1.533 (4)
O1—C2	1.514 (11)	C7—H7A	0.9700
O2—C6	1.335 (3)	C7—H7B	0.9700
O2—C7	1.451 (4)	C8—C11	1.513 (4)
O7—H7C	0.87 (4)	C8—C12	1.515 (5)
N1—C3	1.271 (4)	C9—H9A	0.9600
N1—C1	1.494 (3)	C9—H9B	0.9600
N2—C6	1.275 (3)	C9—H9C	0.9600
N2—C8	1.495 (3)	C10—H10A	0.9600
N2—Ag^ii^	2.120 (2)	C10—H10B	0.9600
C1—C10	1.505 (5)	C10—H10C	0.9600
C1—C9	1.512 (5)	C11—H11A	0.9600
C1—C2	1.518 (12)	C11—H11B	0.9600
C1—C2'	1.574 (19)	C11—H11C	0.9600
C2—H2B	0.9700	C12—H12A	0.9600
C2—H2C	0.9700	C12—H12B	0.9600
C2'—H2'A	0.9700	C12—H12C	0.9600
C2'—H2'B	0.9700		
			
N1—Ag—N2^i^	171.07 (8)	H4B—C4—H4C	107.7
O4—Cl—O6	109.1 (3)	C6—C5—C4	113.4 (2)
O4—Cl—O5	109.7 (2)	C6—C5—H5B	108.9
O6—Cl—O5	109.7 (2)	C4—C5—H5B	108.9
O4—Cl—O3	107.6 (2)	C6—C5—H5C	108.9
O6—Cl—O3	107.9 (2)	C4—C5—H5C	108.9
O5—Cl—O3	112.8 (2)	H5B—C5—H5C	107.7
C3—O1—C2'	107.2 (9)	N2—C6—O2	117.1 (2)
C3—O1—C2	103.9 (4)	N2—C6—C5	127.5 (2)
C2'—O1—C2	28.7 (9)	O2—C6—C5	115.4 (2)
C6—O2—C7	106.1 (2)	O2—C7—C8	104.8 (2)
C3—N1—C1	108.2 (2)	O2—C7—H7A	110.8
C3—N1—Ag	127.74 (19)	C8—C7—H7A	110.8
C1—N1—Ag	124.10 (17)	O2—C7—H7B	110.8
C6—N2—C8	108.3 (2)	C8—C7—H7B	110.8
C6—N2—Ag^ii^	125.88 (19)	H7A—C7—H7B	108.9
C8—N2—Ag^ii^	125.67 (16)	N2—C8—C11	109.3 (2)
N1—C1—C10	110.4 (3)	N2—C8—C12	110.2 (3)
N1—C1—C9	109.0 (3)	C11—C8—C12	110.9 (3)
C10—C1—C9	110.3 (3)	N2—C8—C7	101.2 (2)
N1—C1—C2	101.7 (4)	C11—C8—C7	112.3 (3)
C10—C1—C2	104.8 (4)	C12—C8—C7	112.6 (3)
C9—C1—C2	120.1 (5)	C1—C9—H9A	109.5
N1—C1—C2'	100.6 (8)	C1—C9—H9B	109.5
C10—C1—C2'	128.8 (9)	H9A—C9—H9B	109.5
C9—C1—C2'	95.9 (9)	C1—C9—H9C	109.5
C2—C1—C2'	27.2 (9)	H9A—C9—H9C	109.5
O1—C2—C1	101.7 (6)	H9B—C9—H9C	109.5
O1—C2—H2B	111.4	C1—C10—H10A	109.5
C1—C2—H2B	111.4	C1—C10—H10B	109.5
O1—C2—H2C	111.4	H10A—C10—H10B	109.5
C1—C2—H2C	111.4	C1—C10—H10C	109.5
H2B—C2—H2C	109.3	H10A—C10—H10C	109.5
O1—C2'—C1	104.3 (12)	H10B—C10—H10C	109.5
O1—C2'—H2'A	110.9	C8—C11—H11A	109.5
C1—C2'—H2'A	110.9	C8—C11—H11B	109.5
O1—C2'—H2'B	110.9	H11A—C11—H11B	109.5
C1—C2'—H2'B	110.9	C8—C11—H11C	109.5
H2'A—C2'—H2'B	108.9	H11A—C11—H11C	109.5
N1—C3—O1	116.6 (3)	H11B—C11—H11C	109.5
N1—C3—C4	127.4 (3)	C8—C12—H12A	109.5
O1—C3—C4	116.0 (2)	C8—C12—H12B	109.5
C3—C4—C5	113.6 (2)	H12A—C12—H12B	109.5
C3—C4—H4B	108.9	C8—C12—H12C	109.5
C5—C4—H4B	108.9	H12A—C12—H12C	109.5
C3—C4—H4C	108.9	H12B—C12—H12C	109.5
C5—C4—H4C	108.9		

Symmetry codes: (i) *x*, −*y*, *z*+1/2; (ii) *x*, −*y*, *z*−1/2.

### Hydrogen-bond geometry (Å, º)

**Table:**

*D*—H···*A*	*D*—H	H···*A*	*D*···*A*	*D*—H···*A*
O7—H7*C*···O3	0.87 (5)	2.06 (5)	2.926 (4)	175 (4)
C4—H4*C*···O7^iii^	0.97	2.47	3.379 (4)	156
C5—H5*B*···O5^ii^	0.97	2.39	3.289 (5)	153

Symmetry codes: (ii) *x*, −*y*, *z*−1/2; (iii) −*x*, −*y*, −*z*+1.
